# Longitudinal evaluation of anti-SARS-CoV-2 neutralizing antibody levels in 3-dose homologous (mRNA-1273- mRNA-1273- BNT162b2) vaccinated kidney transplant population: 18-month follow-up

**DOI:** 10.1016/j.ijregi.2025.100767

**Published:** 2025-09-22

**Authors:** Kankanamalage Ridma Prasadini Karunathilake, Roshan Athula Kumara, Amali Karunathilaka, Abdul Wahid Mohamed Wazil, Nishantha Nanayakkara, Chandana Keerthi Bandara, Rajitha Asanga Abeysekera, Faseeha Noordeen, Indika Bandara Gawarammana, Champa Neelakanthi Ratnatunga

**Affiliations:** 1 Department of Microbiology, Faculty of Medicine, University of Peradeniya, Kandy, Sri Lanka; 2 Nephrology Unit, National Hospital Kandy, Kandy, Sri Lanka; 3 Department of Medicine, Faculty of Medicine, University of Peradeniya, Kandy, Sri Lanka

**Keywords:** Kidney Transplant, COVID-19, Homologous vaccination, Seroconversion, Neutralizing Antibody Levels, Longitudinal Follow-up

## Abstract

**Objectives:**

This study aimed to assess the immune response to a three-dose primary series of COVID-19 vaccination in kidney transplant recipients (KTRs), a population vulnerable to infection due to immunosuppression.

**Methods:**

This study was a longitudinal evaluation of neutralizing antibody (nAB) dynamics in 43 KTRs in a lower-middle-income setting receiving a three-dose homologous (messenger RNA [mRNA]-1273-mRNA-1273- BNT162b2) vaccination against COVID-19. Samples were obtained at time points (TP) as follows: TP0, pre-vaccination; TP1, 1-month post-first dose (mRNA-1273); TP2, 1-month post-second dose (mRNA-1273); TP3, 4 months post-second dose; TP4, 2 weeks post-third dose (BNT162b2); TP5, 5 months post-third dose; and TP6, 12 months post-third dose. Anti-SARS-CoV-2 nAB were detected using the Genscript cPass^TM^ pseudoviral neutralization kit. Demographic and clinical details were obtained through interviewer-administered questionnaires.

**Results:**

Pre-vaccination serum analysis showed n = 7 KTRs had prior COVID-19 infection, classified as ‘infected + vaccinated,’ while others were ‘vaccinated.’ Both groups were similar in age (41.7 years vs 46.7 years, *P* = 0.2383), gender, and transplant characteristics. Seroconversion and mean/ median antibody level (MAB) in the vaccinated and infected + vaccinated KTRs were: TP1, 8.3% vs 100% (*P* <0.001), MAB = 64.3 IU/mL vs 1424 IU/mL (*P* = 0.0167); TP2, 52.7% vs 100% (*P* = 0.0194), MAB = 175 IU/mL vs 2790 IU/mL (*P* <0.0001); TP3, 100% vs 100%, MAB = 106IU/mL vs 2153 IU/mL (*P* = 0.0002); TP4, 100% vs 100%, MAB = 736 IU/mL vs 2152 IU/mL (*P =* 0.0307); and TP6, 100% vs 100%, MAB > 2565 IU/mL vs > 3028 IU/mL (*P* = 0.5238). No factors were associated with seroconversion or MAB.

**Conclusions:**

KTRs receiving a three-dose mRNA COVID-19 vaccine regimen maintained strong nAB levels at 1-year follow-up, with comparable antibody levels seen between KTRs with prior infection + vaccination and vaccination alone.

## Highlights

• Kidney transplant recipients with prior COVID-19 had high neutralizing antibody levels throughout an 18-month follow-up.
• Kidney transplant recipients without prior infection showed lower responses and de novo seroconversion.
• Both groups had similar, high neutralizing antibody levels at 12 months post-third dose.

## Introduction

Kidney transplant recipients (KTRs) were among the most vulnerable during the early COVID-19 pandemic, facing infection rates of 20-30% and alarmingly high mortality [1,2]. Their immunosuppressed state not only made them prone to severe disease but also prolonged viral clearance, increasing the risk of viral mutations and transmission [3]. Although prioritized for vaccination, early studies showed weak antibody responses after two messenger RNA (mRNA) vaccine doses, with only 22-36.4% of KTRs developing anti-SARS-CoV-2 immunoglobulin (Ig)G antibodies after two doses of BNT162b2 [4,5] and only 48-65% after two doses of mRNA-1273 [6,7]. Even when formed, antibodies waned rapidly over a 6-month period [8]. Given the low antibody levels and almost absent T-cell responses seen in seroconverted KTRs [9], additional vaccine doses for these vulnerable individuals were recommended by the World Health Organization (WHO) [10]. Recent studies confirmed the improved response to the third dose of the mRNA vaccine in achieving sustained seroconversion and antibody levels in KTRs [11,12].

Our study focused on a cohort of KTRs in Sri Lanka, a lower-middle-income country that went through four waves of COVID-19 from March 2020 to December 2022, with a consequent economic crisis and social upheaval that severely affected all aspects of life. However, the national vaccine program provided priority vaccination for patients on dialysis and KTRs from July 2021. COVID-19-related mortality in this patient group was 33.3% before vaccination in Sri Lanka [13]. Patients were given a three-dose homologous vaccine combination of mRNA-1273, mRNA-1273, and BNT162b2, based on in-country vaccine availability. We evaluated the longitudinal neutralizing antibody (nAB) dynamics throughout the completion of the vaccination and continued a 1-year follow-up after the third dose while analyzing the factors associated with seroconversion. The objective was to study the longevity and level of neutralizing humoral responses in these patients.

## Materials and methods

### Study population

This longitudinal study was carried out between July 20, 2021, and December 30, 2022, up to 1 year following administration of the third vaccine dose to KTRs. Initially, 43 KTRs aged over 18 years who attended the Nephrology Unit of the National Hospital Kandy, the largest kidney transplant center in the country, were recruited after obtaining informed written consent for participation in the longitudinal study. In addition, verbal consent was obtained at each longitudinal sampling point following the initial sampling. Any patient who wished to withdraw at any stage was free to do so according to the approved study protocol (Supplementary Figure 1). Ethical clearance (2021/EC/14) was obtained from the Ethics Review Board of the Faculty of Medicine, University of Peradeniya, Sri Lanka.

**Figure 1:**
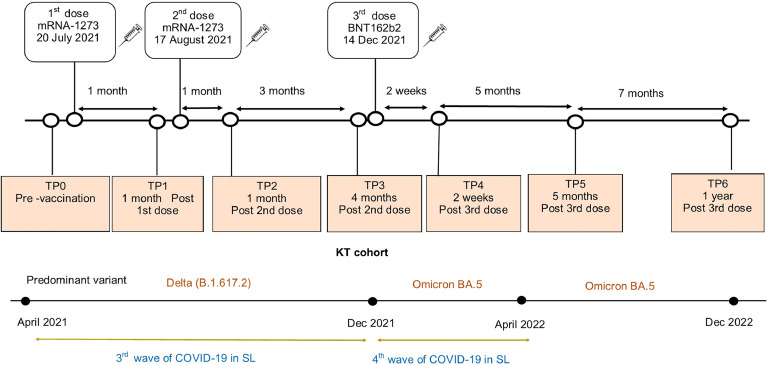
Timeline of vaccination and sampling time points. The timeline shows the timing of vaccination and sampling time points of the patient cohort along with the timing of concurrent waves of COVID-19. KT, kidney transplant; mRNA, messenger RNA; TP, time point.

The KTRs were given two doses of mRNA-1273, 1 month apart, and a third vaccine dose of BNT162b2, 4 months later, to complete the primary series. Serum sample collection was done at seven time points in relation to vaccine administration as shown in Figure 1: pre-vaccination (TP0), 1-month post-first dose (TP1), 1 month post-second dose (TP2), 4 months post-second dose (TP3), 2 weeks post-third dose (TP4), 5 months post-third dose (TP5), and 1 year post-third dose (TP6).

Sociodemographic data, history of symptoms suggestive of COVID-19 infection or COVID-19 confirmed by reverse transcription polymerase chain reaction (RT-PCR) or antigen detection (testing was freely available at the study site throughout the study period), and vaccine adverse events related to each vaccine dose were collected from each participant at each time point using an interviewer-administered questionnaire. Furthermore, the duration of transplant, history of transplant rejection, and patients on immunosuppressive drugs (prednisolone, mycophenolate mofetil [MMF], tacrolimus, cyclosporin, everolimus) were documented.

Throughout follow-up, from TP2 to TP3, sample numbers had to be restricted due to limited test availability, while during subsequent time points, dropouts due to refusal or inability to participate because of logistical or economic reasons were noted. A summary of participant recruitment and follow-up is shown in Supplementary Figure 1. When sampling restriction was required, participants who agreed to continued participation for long-term testing, planned to take the third dose, and were readily contactable were selected.

Blood samples were collected to detect SARS-CoV-2 neutralizing antibodies using the Genscript cPass™ Neutralization Antibody Detection Kit (GenScript USA/Nanjing GenScript, RUO Version 1.0). Serum was stored at -80 °C and tested per the manufacturer’s instructions. Percentage inhibition (calculated based on manufacturer instructions) was obtained using the surrogate viral neutralization assay. Seroconversion was defined as a percentage inhibition value above a cutoff of 30% neutralization, while the level of antibody was equated to the percent neutralization calculated. Additional antibody quantification (IU/mL) followed the WHO guidelines [14] and a published conversion algorithm [15]. Values > 97.59% inhibition (> 3028 IU/mL) required dilution and retesting for accurate IU conversion; this was done for only eight of 18 samples (from TP1 and TP2) due to financial constraints. TP6 samples from ‘vaccinated’ (n = 8) and ‘infected + vaccinated’ (n = 2) groups could not be retested. For visualization, these sample values were plotted as >3028 IU/mL and analyzed statistically as 3028 IU/mL. The conversion algorithm was log-scaled; therefore, mean values are expressed as geometric mean with an SD factor. Results are therefore expressed as seroconversion percentage (the number of individuals with antibody levels above the defined cutoff), mean/median antibody level (MAB, the midpoint estimate of percentage neutralization of the positive samples within the group), and the equivalent geometric mean antibody unit level as IU/mL.

### Statistical analysis

Statistical analysis was carried out using GraphPad Prism (8.4.2. 679, 2020). Percentage neutralization was compared using the Mann–Whitney U test (MWU), and the association between categorical variables was evaluated using the chi-square test [16].

## Results

Initially, n = 43 KTRs were recruited, in whom pre-vaccination samples (TP0) revealed seven (7/43) with positive neutralizing antibodies despite having no history of suggestive symptoms or RT-PCR test positivity. This patient subset was categorized as ‘infected + vaccinated’ KTRs. The pre-vaccination seronegative KRTs (n = 36) were categorized as ‘vaccinated’ KTRs. During follow-up, none of the participants developed clinical symptoms suggestive of, or RT-PCR confirmed, SARS-CoV-2 infection. The number of serum samples in each group at each time point is shown in Supplementary Figure 1.

### Demographic characteristics

#### Age and gender distribution

Vaccinated KTRs and infected + vaccinated KTRs were similar in age (41.7 years [SD ± 11.2]) vs 46.7 years [SD ± 11.2], MWU, *P* = 0.2383), as well as gender distribution (chi-square, df = 2, *P* = 0.8429), with both groups having a male majority. Duration of transplant, history of transplant rejection, and the number of KTRs on different immunosuppressive drugs are shown in Table 1. The mean duration of transplant in the vaccinated KTRs (56.6 months) was similar (MWU, *P* = 0.3108) to that of infected + vaccinated KTRs (63.5 months), with an overall range of 10 months to over 15 years, while nearly half of the total KTRs had undergone kidney transplant 2-5 years ago. Prednisolone (vaccinated: 100%, infected + vaccinated: 100%), MMF (vaccinated: 97.6%, infected + vaccinated: 85.7%, chi-square, *P* = 0.1628), and Tacrolimus (vaccinated: 94.4%, infected + vaccinated: 100%, chi-square, *P* >0.9999) were the most common immunosuppressive drugs that the patients in both groups were on, in similar frequency. A summary of immunosuppressant medication doses and patient numbers is provided in Supplementary Table 1. KTRs in both groups had been receiving a maximum of three immunosuppressive agents. The history of transplant rejection was similar in both groups (vaccinated: 63.8% [23/36], infected + vaccinated: 71.4% [5/7], chi-square, *P* = 0.7017). All the KTRs were vaccinated with a three-dose hepatitis B vaccine series, though the seroconversion status is unconfirmed.

**Table 1:** Kidney transplant recipient characteristics.

Characteristic	Value/ Frequency		*P*-value
	Vaccinated	Infected + Vaccinated	
Age			
Mean (SD)/median (IQR)	41.7 (11.2) years/ 40.5 (32.5-50)	46.7 (11.2) years/ 50 (39-55)	*P* = 0.2383^a^
Range	23-69 years	26-60 yeas	
Gender			
Male	75% (27 /36)	71.4% (5 /7)	*P* = 0.8429^b^
Female	25% (9 /36)	28.6% (2 /7)	
Transplant duration			
Mean (SD)/median (IQR)	56.6 (43) months/ 48.5 (26-77)	63.5 (35) months/ 57 (36-77)	*P* = 0.3108
Range	10-190 months	32-132 months	
<2 years	22.2% (8 /36)	0	
2-5 years	41.6% (15 /36)	57.2% (4 /7)	
5-10 years	25% (9 /36)	28.6% (2 /7)	
> 10 years	11.2% (4 /36)	14.2% (1 /7)	
Patients with a history of transplant rejection	63.8% (23 /36)	71.4% (5 /7)	*P* = 0.7017^b^
**Patients on immunosuppressive drugs**			
Prednisolone	100% (36 /36)	100% (7 /7)	
MMF	100% (36 /36)	85.7% (6 /7)	*P* = 0.1628^b^
Tacrolimus	94.4% (34 /36)	100% (7 /7)	*P* >0.9999^b^
Cyclosporin	2.7% (1 /36)	0	
Everolimus	11.1% (4 /36)	14.2% (1 /7)	*P* >0.9999^b^

a Mann-Whitney U test.

b Chi square/Fisher's exact test.

#### Longitudinal antibody dynamics following two doses of mRNA-1273 and a third dose with BNT162b2 in vaccinated KTRs

**Figure 2:**
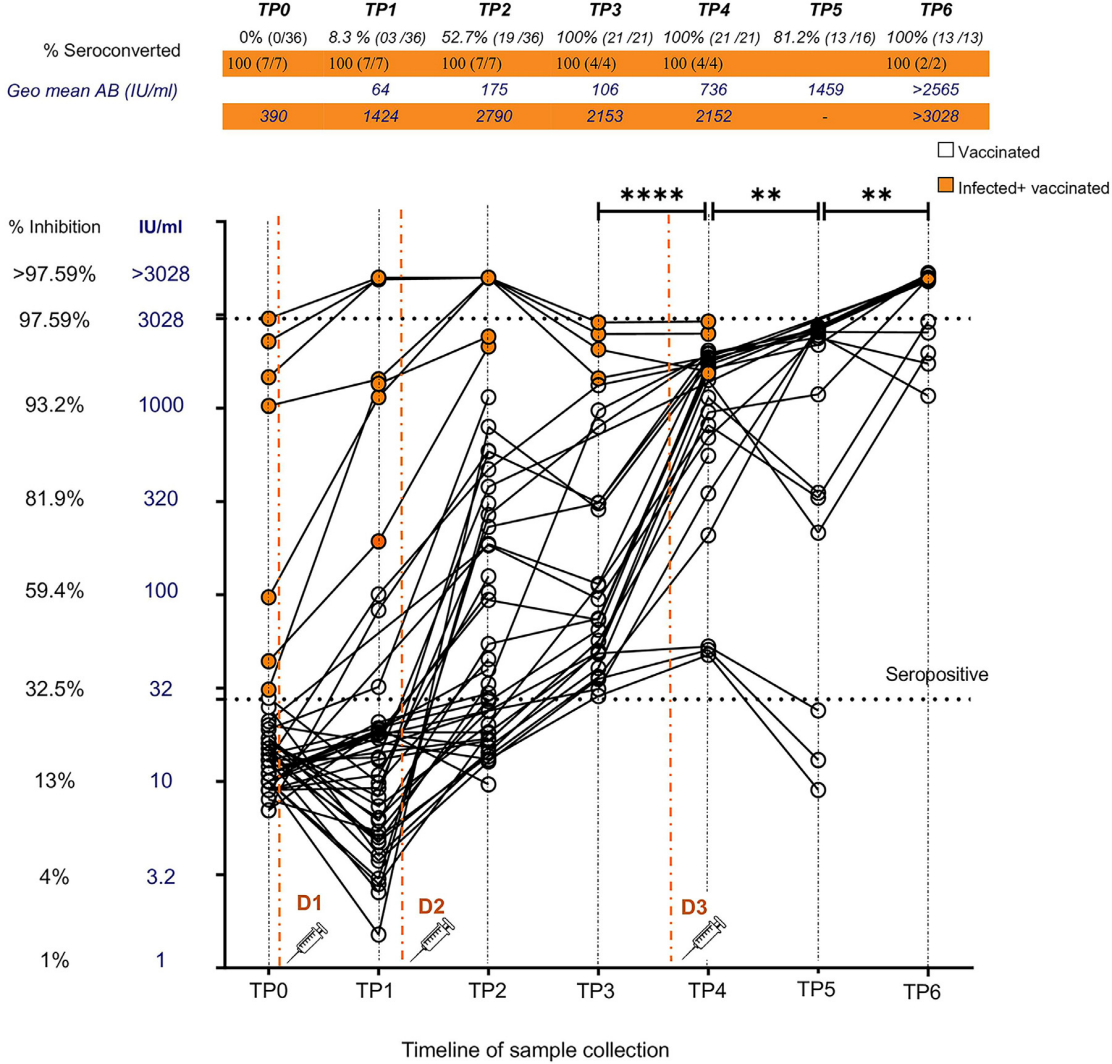
Longitudinal nAB dynamics following two doses of mRNA-1273 and a third dose of BNT162b2. Dot plot shows neutralizing antibody dynamics in KTRs with natural infection + vaccination (orange circles) and vaccine only (open circles) at the time points of pre-vaccination (TP0), 1-month post first dose (TP1), 2 weeks post second dose (TP2), 4 months post second dose (TP3), 2 weeks post third dose (TP4), 5 months post third dose (TP5) and 12 months post third dose (TP6). The primary Y axis (blue) shows the antibody level in IU/mL, while the secondary Y axis (black) shows the equivalent percentage neutralization value. Seropositivity is designated at >30% (28 IU/mL) (manufacturer's standard). Data points plotted at >3028 IU/mL (>97.59% inhibition) indicate the individuals with antibody levels that range between 3878-29133 IU/mL. A significant increase in antibody levels in ‘vaccinated KTRs’ was seen after third dose vaccination (TP3 to TP4) and during the 1-year follow-up period (TP4 to TP5; TP5 to TP6). Infection + vaccinated KTRs showed persistently high nAB levels throughout follow-up. Red dashed lines indicate the time points of vaccine administration. (D1– first dose, D2– second dose, D3- third dose). Statistically significant difference in mean/ median antibody level (Mann-Whitney U test) denoted by P <0.05-*, P <0.01- **, P <0.001 - ***, P <0.0001 - ****. nAB, neutralizing antibody; TP, time point.

Neutralizing antibody dynamics in both KTR groups (vaccinated and infected + vaccinated) following two doses of mRNA-1273 and a third dose with BNT162b2 are shown in Figure 2. All patients in the ‘vaccinated’ group were seronegative prior to vaccination. The percentage seropositive at TP1, 1 month after the first dose, was 8.3% (03/36) with a moderate median antibody level (MAB) of 54.85% neutralization (interquartile range [IQR]: 32.59-59.49%), which corresponds to 64.37 IU/mL (SD factor: 1.84). At TP2, 2-4 weeks following the second dose, seropositivity significantly increased (chi-square, *P* <0.001) to 52.7% (19/36) while MAB increased (MWU, *P* = 0.1325) to 72.90% neutralization (IQR: 44.70-86.84%), correlating to a unit value of 175.40 IU/mL (SD factor: 3.09). At TP3, 4 months following the second dose, seropositivity significantly increased again (chi-square, *P* <0.001) to 100% (21/21), though MAB dropped somewhat (MWU, *P* = 0.2055) to 51.96% neutralization (IQR: 39.84-80.90%), with a unit value 106.6 IU/mL (SD factor: 3.25). The changes in antibody levels (MAB and unit value as IU/mL) were not statistically different between any of these initial time points. However, a deeper look revealed that while all vaccinated KTRs had become seropositive by TP3 (21/21), the 52.4% who were seronegative at TP2 (11/21) showed a significant increase in nAB levels at TP3 (mean % inhibition/IU/mL [95% confidence interval]: 21% [18.4-24.3%]/17.5 IU/mL [14.6-20.9 IU/mL] vs 41.2% [35.1%-47.3%]/46.7 IU/mL [36-60.4 IU/mL], paired *t* test, *P* <0.0001), while the 47.6% who were seropositive at TP2 (10/21) showed no significant difference in antibody levels (70.5% [56.6-84.3%]/195 IU/mL [93.8-404.7 IU/mL] vs 74.7% [62.6-86.9%]/264.6 IU/mL [122-574 IU/mL], paired *t* test, *P* = 0.5488) at TP3.

Two weeks following the administration of the BNT162b2 vaccine as the third dose (TP4), seropositivity remained constant at 100% (21/21), while the MAB significantly increased (MWU, *P* <0.0001) to 95.08% neutralization (IQR: 85.88-96.05%), with an equivalent unit value of 736.50 IU/mL (SD factor: 3.58). At TP5, 5 months after dose 3, seropositivity significantly reduced (chi-square, *P* <0.05) to 81.25% (13/16), though the MAB of the seropositive KTRs increased significantly (MWU, *P* = 0.0065) to 97.06% neutralization (IQR: 88.74-97.29%), 1459 IU/mL (SD factor: 2.58). A deeper analysis showed that 76.9% (10/13) of KTRs who were seropositive at TP5 showed increasing nAB levels compared to TP4 (95.7% [IQR: 88.7-96.3%]/1062 IU/mL [595-1896 IU/mL] vs 97.2% [IQR: 96.9-97.3%]/2363 IU/mL [1973-2829 IU/mL], Wilcoxon test, *P* = 0.0020), while 23% (3/13, seropositive at TP5) showed a decreasing trend without a significant difference in nAB levels (94% [IQR: 91.8-95%]/1095 IU/mL [544.6-2200 IU/mL] vs 82.4% [75.4- 83.2%]/292.6 IU/mL [150-570 IU/mL], Wilcoxon test, *P* = 0.2500). Three KTRs (18.7% of the total tested at TP5) seroreverted at 5 months post-third dose. As shown in Figure 2, these were KTRs who showed persistently low antibody levels, despite late seroconversion 4 months after the second dose. Other KTRs who showed late seroconversion after the second dose (n = 8) showed a significant boost in antibody levels following the third dose. There was no significant difference in terms of age, gender, duration of transplant, type of immunosuppressant, or history of transplant rejection between the KTRs who had persistently low nAB responses and subsequently seroreverted, compared to the other low responders who later showed good responses to the third dose.

Twelve months following the third dose (TP6), 100% (13/13) seropositivity was observed, while MAB significantly increased (MWU, *P* = 0.0075) up to 97.85% neutralization (IQR: 96.77-98.01%), with a very high equivalent unit value of 2565 IU/mL (SD factor:1.35). While 61.5% (n = 8) of patients showed a consistent increase in antibody levels, 15% (n = 2) of patients who showed an initial drop at 5 months also showed a subsequent increase at 1-year follow-up. A lack of 1-year data on the KTRs who seroreverted at 5 months is noted here. A comparison of % seroconverted and MAB level (geometric mean IU/mL and % inhibition) between each consecutive time point in the vaccinated KTRs is shown in Supplementary Table 2.

#### Longitudinal antibody dynamics in ‘infected + vaccinated’ KTRs

Prior to vaccination (TP0), seven KTRs found to be positive for nAB had a median antibody level of 93.39% neutralization (IQR: 39.61-96.85%), which is equivalent to a unit value of 390 IU/mL (SD factor:7.11). This initial MAB did not exhibit a significant change after the first dose (TP1: 95.12%, IQR: 94.02-97.74% / 1424 IU/mL, SD factor: 2.66, MWU, *P* = 0.1649) or second dose at either TP2 (97.71%, IQR: 97.02-97.75%/ 2790 IU/mL, SD factor: 1.15, MWU, *P* = 0.1183) or TP3 (96.82%, IQR: 95.51-97.38%/ 2153 IU/mL, SD factor: 1.35, MWU, *P* = 0.0727), though an increasing trend was seen. The range of nAB in dilution-tested samples was 3878-29133 IU/mL. Sample collection following the third dose was limited; however, collected samples revealed no significant change in nAB levels following the third dose by TP4 (96.66%, IQR: 95.7-97.4%/ 2152 IU/mL, SD factor: 1.32, MWU, *P* = 0.8857). While logistic difficulties prevented sample collection at TP5, sustained high antibody levels were seen at 1-year follow-up (TP6: >97.98%, IQR: 97.92-98.04%, >3028 IU/mL, SD factor: 1, MWU, *P* = 0.1333), where both samples collected had nAB levels requiring dilution retesting.

### Comparison between the ‘vaccinated’ group and the ‘infected + vaccinated’ group

The MAB and seroconversion rate in KTRs with known natural infection + vaccination and vaccinated KTRs at each time point were compared (Table 2). The naturally infected KTRs maintained 100% seroconversion throughout the course of follow-up, which was significantly higher than that of the vaccinated KTRs group at TP1 and TP2. nAB levels were also consistently higher compared to the infection-naïve group throughout the period starting from 1 month post-first dose (TP1) to 2 weeks post-third dose (TP4). Both groups showed very high MAB at 1 year post-third dose. Statistical comparison could not be performed at TP5 due to a lack of samples in the ‘infected + vaccinated’ group.

**Table 2:** Comparison between ‘vaccinated’ KTRs and ‘infected + vaccinated’ KTRs - percentage seroconversion and MAB.

	D1		D2		D3	
	Measure	TP1	TP2	TP3	TP4	TP6
		1 mo post	1 mo post	4 mo post	2 wks post	12 mo post
		first dose	second dose	second dose	third dose	third dose
Infected +	% Seroconverted	100%	100%	100%	100%	100%
Vaccinated	§MAB geo.mean IU/ml	1424	2790	2153	2152	3028
KTRs	(%inhibition)	(95.12%)	(97.71%)	(96.82%)	(96.66%)	(97.98%)
Vaccinated KTRs	% Seroconverted	8.3%	52.7%	100%	100%	100%
	§MAB geo.mean IU/ml	64.37	175.4	106.6	736.5	2565
	(%inhibition)	(54.85%)	(72.90%)	(51.96%)	(95.08%)	(97.85%)
*P* value	Ϯ*Chi sq*	*p<0.0001*	*p=0.0194*	p>0.9999	p>0.9999	p>0.9999
	¶*MWU*	*p=0.0167*	*p < 0.0001*	*p=0.0002*	*p =0.0307*	*p*=0.5238
	*(MWU)*	(*p=0.0167*)	*(p < 0.0001)*	*(p=0.0002)*	*(p =0.0307)*	(*p*=0.2286)

§ MAB- mean/ median antibody level of seropositive at each time point. Represented as geometric mean (geo.mean) in International Units (IU/ml) and as mean percentage inhibition (% inhibition).

Ϯ Chi sq test was performed to compare the % seroconverted between vaccinated and infected+ vaccinated groups at each time point.

¶ Mann Whitney U (MWU) test was performed to compare the MAB levels of seropositives between vaccinated and infected+ vaccinated groups at each time point in both international units (IU/ml). Comparison of % inhibition between each consecutive timepoint is shown within brackets. (Fishers exact test used when sample size <5). Significance at p<0.05

#### Factors associated with seroconversion and antibody level

None of the demographic or clinical factors, such as duration since kidney transplant, history of transplant rejection, or type of immunosuppressive therapy, were associated with seropositivity or neutralizing antibody level in this study.

## Discussion

In this study, we demonstrate the immune response in terms of nAB dynamics to a homologous three-dose SARS-CoV-2 mRNA vaccine regime (mRNA-1273-mRNA-1273-BNT162b2) in a cohort of KTRs over a period of 18 months, up to 1 year post-third dose with no further boosters. To the best of our knowledge, this is the first study on nAB levels following three-dose mRNA vaccination, providing 1-year follow-up data in KTRs.

We show a seroconversion rate of 52.7% with an MAB of 175.4 IU/mL after two doses of mRNA-1273, in infection-naïve KTRs, similar to the seroconversion (42% -58%) and MAB (25 BAU/mL-142.1 U/mL) observed in several other studies on solid organ transplant and KTRs (two doses mRNA vaccine) [17,18]. A systematic review and meta-analysis of 44 studies involving 5892 KTRs indicated that the overall seroconversion rate (the percentage of nAB, anti-SARS-CoV-2 spike RBD, or either IgG or IgA anti-spike protein that indicated above-normal quantification results) following two doses of mRNA-1273 vaccine in KTRs was 39.2% [19]. Local data on pre-vaccination seroprevalence in KTRs are not available. However, only 8.3% of our cohort were seropositive 1 month after the first dose, which is almost identical to the seroprevalence reported in unvaccinated KTRs in a Brazilian cohort (8.2%) [20]. This indicates that the subsequent robust seroconversion rates were predominantly driven by repeated vaccination itself.

Seroconversion rates calculated will vary with the type of assay used (anti-S1 IgG/ IgA vs nAB, etc.), assay type, its inherent detection limit, and positivity cutoff, as well as the time post-second dose at which sampling was performed, as antibody response patterns are not uniform in these immunosuppressed individuals. Slow humoral responses and delayed seroconversion are known phenomena in transplant recipients [21].

A 6-month follow-up study of two-dose vaccinated KTRs showed that among the 57% who were anti-RBD positive, 54.8% had decreasing, 33.7% stable, and 11.5% increasing antibody levels compared to the 2-month time point, while 5.8% showed de novo seroconversion [22].This delayed or variable humoral response was unique to KTRs and not observed in other kidney disease patients, suggesting immunosuppression-related effects. Similar patterns are seen with influenza [23] and COVID-19 [24] vaccines, and our data also reflect this. At 4 months post-second dose, 52.4% of seropositive KTRs showed delayed seroconversion, possibly due to de novo responses or viral exposure during follow-up. The lack of cross-neutralizing antibodies providing protection from variants, as well as very poor T-cell responses after two-dose vaccination, has also been shown [21,22]. These findings provide the basis for the persistent risk of severe disease and high mortality (5-10%) in KTRs who received only two vaccine doses [25,26].

Although a third dose was recommended 6 months after the second dose and vaccines were available, uptake was low due to travel costs, fuel shortages, and other consequences of a severe economic downturn. Only 21 of 43 (48.8%) KTRs in the study group received it. A significant increase in nAB levels was observed post-third dose, aligning with global data showing improved seroconversion and MAB with 3-dose mRNA vaccine schedules [11,27,28], with 27% of the seropositives achieving anti-spike antibody levels greater than 4160 AU/mL, while that value was as high as 92.9% in healthy controls [28].

At 5 months post-third dose, seropositivity decreased, with a similar pattern of reduced antibody levels and sero-reversion in 18.7% of patients. This was observed in three patients who showed late seroconversion following dose two, with persistently low nAB levels, which subsequently fell below the positivity cutoff. However, overall, a significant increase in antibody level was noted, a trend that continued at 1-year follow-up. Other studies on three-dose-vaccinated solid organ transplant patients [21] have shown similar excellent responses to third-dose vaccination (73%), with antibody levels reaching levels comparable to healthy controls, with better, albeit suboptimal neutralization of variants. KTR-specific data show 49.5% of post-second dose seronegatives developing de novo seroconversion approximately 1 month after dose three [29].

Our data suggest antibody levels may continue rising for up to a year, though it is unclear whether this reflects a delayed vaccine response or environmental exposure. From December 2021 to April 2022 (fourth COVID-19 wave), Omicron BA.5 was prevalent, and TP5/TP6 sampling followed. None of the cohort reported symptoms or tested positive via RT-PCR or antigen tests. Anti-nucleocapsid testing was not done due to funding limitations, and its sensitivity is limited in vaccinated individuals [30]. As the GenScript sVNT targets wild-type RBD, Omicron likely had minimal impact on nAB levels [31]. Thus, responses likely reflect vaccination or early infections, though later variants may have had a mild boosting effect.

Vaccination in those with previous SARS-CoV-2 infection, despite all infections being asymptomatic in this cohort, resulted in higher levels (2790 IU/mL) of immunity after just two doses of COVID-19 vaccination, and the nAB levels were equivalent to those acquired by the infection-naïve group after three vaccine doses (2565 IU/mL). This is similar to data reported by a UK study, which indicates that KTRs with a history of SARS-CoV-2 infection show significantly higher anti-S IgG compared with infection-naïve KTRs following each vaccine in the four-dose COVID-19 vaccine series [29]. Moreover, breakthrough infection after at least three doses of vaccination strengthened and expanded immunity in KTRs, with a significant increase in anti-RBD antibody levels and maturation of vaccine-driven B cell responses, providing cross-neutralizing antibodies while expanding vaccine-driven T-cell responses, indicating an additional boost to vaccine-induced protection in this group [32]. Collectively, these results indicate that natural infection, either prior to vaccination or post-vaccination, provides a boost in the strength and breadth of protective immunity, and a continuing increase in post-vaccination nAB levels could indicate subclinical infection.

In terms of variant neutralization capability and T-cell responses, it has been shown that 100% of the seroconverted KTRs after two doses of COVID-19 vaccines had neutralization capacity against B.1.1.7, while only 64% and 67% showed neutralization against B.1.351 and B.1.617.2, respectively [33].

A systematic review of 37 studies with 3429 KTRs showed de novo seroconversion after third and fourth vaccine doses ranged from 32% to 60% and 25% to 37%, respectively, while variant-specific neutralization ranged between 59% and 70% for Delta and 12% to 52% for Omicron [34]. This evidence highlights the possibility of at least partial protection from SARS-CoV-2 variants in KTRs upon completion of three doses (even in KTRs who showed delayed humoral response), with the neutralization capacity being increased with subsequent booster doses. The longevity of these responses is not yet known, as most studies are based on sampling done within 1-3 months post-vaccination. The importance of our data, though small in scale, is in showing the long-term maintenance of nAB levels in these vulnerable patients, particularly in a resource-limited setting.

In this cohort, demographic and clinical factors such as time post-transplantation, rejection history, and immunosuppressant dose were not associated with seroconversion. However, meta-regression showed that low antibody response was associated with high-dose MMF/mycophenolic acid, belatacept, and anti-CD25 therapy, while tacrolimus use correlated with higher responses [19]. For third and fourth doses, factors such as older age, lower eGFR, early vaccination post-transplant, high immunosuppression burden, diabetes, and use of multiple immunosuppressant classes were linked to non-seroconversion [29].

Overall, our study shows that a three-dose homologous mRNA vaccination elicits a robust and durable neutralizing antibody-mediated immune response in most KTRs who were available for follow-up. As nAB titers before infection are shown to be predictive of protection from day 40 of infection [35], immunity generated either by vaccination or by natural infection with vaccination in KTRs who received a three-dose COVID-19 vaccination provides comparable long-term protection, which could last up to 1 year post-third dose.

A control group of immunocompetent individuals receiving the same vaccine regimen could not be recruited, so comparable nAB data in healthy individuals are unavailable. However, a parallel study in the same setting, involving hemodialysis (HD) patients and healthy controls (HC) on a different schedule (AZD1222–AZD1222–BNT162b2), provides a reference [36]. Two weeks post-second dose, KTRs showed lower MAB (175 IU/mL) than HD (450 IU/mL, *P* = 0.014) and HC (1940 IU/mL, *P* <0.0001). At 4 months, KTRs still had lower levels (106 IU/mL) than HD (235 IU/mL, *P* = 0.032) and HC (453 IU/mL, *P* <0.0001). In contrast, following the third dose, KTR MAB (736 IU/mL) approached that of HD (1029 IU/mL) and HC (1538 IU/mL). These levels remained elevated at 1 year (KTR > 2565 IU/mL; HD > 1961 IU/mL; HC > 2911 IU/mL).

Loss to follow-up rates were high, particularly during the peak of the third wave of the COVID-19 pandemic in Sri Lanka. Retesting of samples requiring dilutions was performed in only 8 of the 18 individuals with high percentage neutralization, giving antibody levels ranging from 3878 IU/mL to 29133 IU/mL. Ten of 18 samples were from the ‘infected + vaccinated’ group (TP1, TP2), while 8 of 18 were from both groups at TP6. The true values of MAB levels at TP6 are therefore probably much higher than estimated in these calculations. The corollary is that better cross-neutralization of variants and protection from severe infection are likely.

## Conclusion

KTRs receiving a three-dose (mRNA-1273–mRNA-1273–BNT162b2) mRNA vaccine regimen show high nAB responses at one-year follow-up, with comparable antibody levels seen between KTRs with prior infection + vaccination and those who had only vaccination. nAB levels shown to be associated with at least partial variant protection lasting at least 1 year after the third dose indicate robust protection in this patient group.

## Declaration of competing interest

The authors have no competing interests to declare.

## Appendix Supplementary materials

Image, application 1

Image, application 2

Image, application 3

Image, application 4
