# Supplementary material for: Longitudinal evaluation of anti-SARS-CoV-2 neutralizing antibody levels in 3-dose homologous (mRNA-1273- mRNA-1273- BNT162b2) vaccinated kidney transplant population: 18-month follow-up
Source: IJID Reg. 2025 Sep 22;17:100767. doi: 10.1016/j.ijregi.2025.100767 (PMC12549382; doi:10.1016/j.ijregi.2025.100767)
Supplement: Supplementary file 1 [file mmc1.docx]

**Supplementary Figure 1. Participant recruitment in KT cohort.** The flow diagram shows the number of participants recruited, excluded, and lost to follow-up at each time point.
