# Supplementary figures and images for: Longitudinal evaluation of anti-SARS-CoV-2 neutralizing antibody levels in 3-dose homologous (mRNA-1273- mRNA-1273- BNT162b2) vaccinated kidney transplant population: 18-month follow-up
Source: IJID Reg. 2025 Sep 22;17:100767. doi: 10.1016/j.ijregi.2025.100767 (PMC12549382; doi:10.1016/j.ijregi.2025.100767)

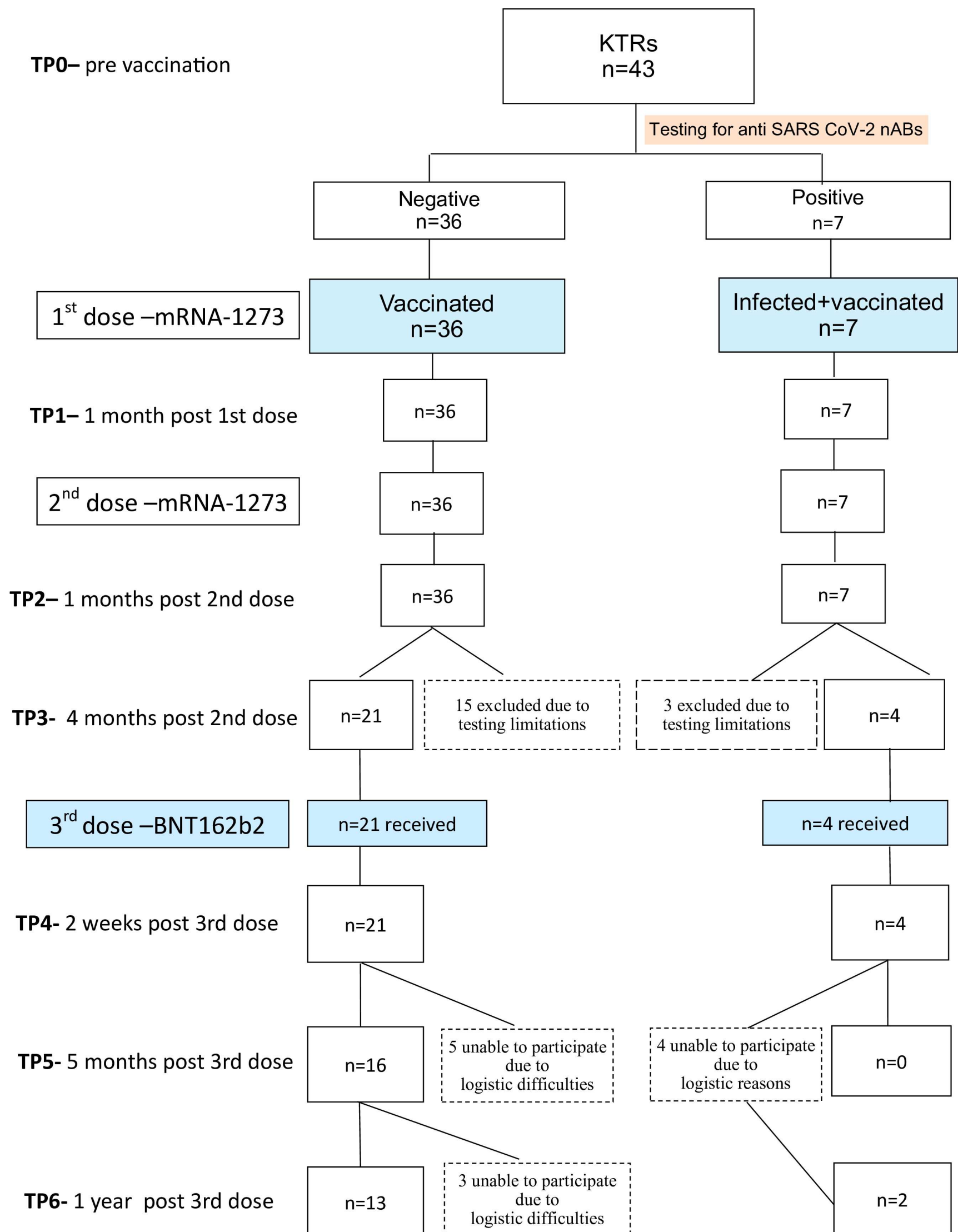

Supplement: Supplementary file 2 [file mmc2.pdf]
