# Supplementary material for: Longitudinal evaluation of anti-SARS-CoV-2 neutralizing antibody levels in 3-dose homologous (mRNA-1273- mRNA-1273- BNT162b2) vaccinated kidney transplant population: 18-month follow-up
Source: IJID Reg. 2025 Sep 22;17:100767. doi: 10.1016/j.ijregi.2025.100767 (PMC12549382; doi:10.1016/j.ijregi.2025.100767)
Supplement: Supplementary file 3 [file mmc3.docx]

**Supplementary Table 1.** **A summary of immunosuppressant medication doses and patient numbers**

| The type of immunosuppressant | Dosage (mg) | Number of KTRs out of total 43 |
| --- | --- | --- |
| Mycophenolate Mofetil (MMF) | 500  750  1000  1500  2000 | 1  2  23  15  1 |
| Tacrolimus | 1  1.5  2  3  4  5  6  7  8 | 1  1  21  6  7  2  1  1  1 |
| Cyclosporin | 75 | 1 |
