# Supplementary material for: Longitudinal evaluation of anti-SARS-CoV-2 neutralizing antibody levels in 3-dose homologous (mRNA-1273- mRNA-1273- BNT162b2) vaccinated kidney transplant population: 18-month follow-up
Source: IJID Reg. 2025 Sep 22;17:100767. doi: 10.1016/j.ijregi.2025.100767 (PMC12549382; doi:10.1016/j.ijregi.2025.100767)
Supplement: Supplementary file 4 [file mmc4.docx]

**Supplementary Table 2. Comparison of percentage seroconverted and mean/ median antibody levels between consecutive time points in Vaccinated KTRs**

|  | | **D1** | |  | | **D2** | |  | | | | | **D3** |  | | | | |
| --- | --- | --- | --- | --- | --- | --- | --- | --- | --- | --- | --- | --- | --- | --- | --- | --- | --- | --- |
|  | Measure | | TP1  1 mo post  First dose | | | | TP2  1 mo post  second dose | | | TP3  4 mo post  Second dose | | | TP4  2 wks post  third dose | | | TP5  5 mo post  third dose | | TP6  12 mo post  Third dose |
| Vaccinated KTR | % Seroconverted  *^a^*MAB geo.mean IU/ml  (%inhibition) | | 8.3%  64.37  (54.85%) | | | | 52.7%  175.4  (72.90%) | | | 100%  106.6  (51.96%) | | 100%  736.5  (95.08%) | | | | 81.25%  1459  (97.06%) | | 100%  2565  (97.85%) |
| *P* value | ^b^Chi sq  *^c^MWU*  *(MWU)* | | | | *p* < 0.001  p>0,05  p>0,05 | | | | *p* <0.05  p>0,05  p>0,05 | | p>0.05  *p < 0.0001*  *(p < 0.0001)* | | | | *p* <0.05  *p* < 0.05  (*p* < 0.05) | | p>0,05  *p* < 0.05  (*p* < 0.05) | |

***^a^***MAB- mean/ median antibody level of seropositive at each timepoint. Represented as geometric mean (geo.mean) in International Units (IU/ml) and as mean percentage inhibition (% inhibition).

***^b^***Chi sq test was performed to compare the % seroconverted between each consecutive timepoint.

***^c^***Mann Whitney U (MWU) test was performed to compare the MAB levels of seropositive between each consecutive timepoint in both International units (IU/ml). Comparison of % inhibition between each consecutive timepoint is shown within brackets. Significance at p<0.05
